# A Compact High-Quality Image Demosaicking Neural Network for Edge-Computing Devices

**DOI:** 10.3390/s21093265

**Published:** 2021-05-08

**Authors:** Shuyu Wang, Mingxin Zhao, Runjiang Dou, Shuangming Yu, Liyuan Liu, Nanjian Wu

**Affiliations:** 1 State Key Laboratory of Superlattices and Microstructures, Institute of Semiconductors, Chinese Academy of Sciences, Beijing 100083, China; wangshuyu@semi.ac.cn (S.W.); zhaomingxin17@semi.ac.cn (M.Z.); dourj@semi.ac.cn (R.D.); yushuangming@semi.ac.cn (S.Y.); nanjian@red.semi.ac.cn (N.W.); 2 Center of Materials Science and Optoelectronics Engineering, University of Chinese Academy of Sciences, Beijing 100049, China; 3 Center for Excellence in Brain Science and Intelligence Technology, Chinese Academy of Sciences, Beijing 100083, China

**Keywords:** image sensor, image demosaicking, convolutional neural network, bayer color filter array, U-Net, edge computing

## Abstract

Image demosaicking has been an essential and challenging problem among the most crucial steps of image processing behind image sensors. Due to the rapid development of intelligent processors based on deep learning, several demosaicking methods based on a convolutional neural network (CNN) have been proposed. However, it is difficult for their networks to run in real-time on edge computing devices with a large number of model parameters. This paper presents a compact demosaicking neural network based on the UNet++ structure. The network inserts densely connected layer blocks and adopts Gaussian smoothing layers instead of down-sampling operations before the backbone network. The densely connected blocks can extract mosaic image features efficiently by utilizing the correlation between feature maps. Furthermore, the block adopts depthwise separable convolutions to reduce the model parameters; the Gaussian smoothing layer can expand the receptive fields without down-sampling image size and discarding image information. The size constraints on the input and output images can also be relaxed, and the quality of demosaicked images is improved. Experiment results show that the proposed network can improve the running speed by 42% compared with the fastest CNN-based method and achieve comparable reconstruction quality as it on four mainstream datasets. Besides, when we carry out the inference processing on the demosaicked images on typical deep CNN networks, Mobilenet v1 and SSD, the accuracy can also achieve 85.83% (top 5) and 75.44% (mAP), which performs comparably to the existing methods. The proposed network has the highest computing efficiency and lowest parameter number through all methods, demonstrating that it is well suitable for applications on modern edge computing devices.

## 1. Introduction

In an ideal color digital camera system, there should be three image sensors that capture the red, green, and blue light component signals of photo images, respectively. However, that is complex and expensive. Now, most color digital cameras normally use an image sensor with a top color filter array (CFA) to capture the intensity of a single color component signal per pixel. There are different types of CFA patterns depending on the manufacturer, such as Bayer CFA, Yamanaka CFA, and HVS-based CFA [1]. Among them, the Bayer CFA pattern is the most widely used one. Because the pixel signal data acquired through CFA constitutes a mosaic-like monochrome image, the RGB color image must be restored by an image processing method called demosaicking [2].

Traditional image demosaicking methods include interpolation-based methods, dictionary-based methods, etc. Malvar [3] and Wang [4] proposed a bilinear interpolation method based on gradient correction, which considers the correlation between color channels. However, they ignore the orientation information, and their results tend to introduce artifacts at the edges of images. Kiku [5,6] proposed a series of residual interpolation-based demosaicking algorithms (RI) and minimized-Laplacian residual interpolation methods based on minimum Laplacian (MLRI); Monno [7] proposed an adaptive residual interpolation algorithm (ARI); Moghadam [8] used a manually constructed dictionary-based method to distinct inter-channel and inter-pixel correlations of natural images. Interpolation-based methods typically take observations of mosaic images’ local properties and utilize correlations between channels. However, those manually extracted features often fail to reconstruct complex structures and introduce many image artifacts, such as zippering, blurring artifacts, false colors, and moiré [9]. Those dictionary-based methods take a longer time to demosaick so are less practical.

Due to the rapid development of deep learning in image processing, demosaicking methods based on CNN have also been proposed in recent years [10,11,12,13,14]. The CNN-based method, which is a kind of data-driven approach, on the other hand, can automatically extract image features and complete the reconstruction process, making them suitable for implementation on some AI accelerators [15,16,17] or vision chips [18,19,20,21]. Although they improve the quality of the demosaicked image markedly, they need to store a large number of the model parameter and cost huge computational time [22]. Several deep learning-based demosaicking methods proposed in recent years focus too much on demosaicking accuracy while ignoring the computational cost of the algorithm. That makes the network unable to meet image sensors’ post-processing real-time requirements. Therefore, CNN-based demosaicking methods should balance computational cost and demosaicking accuracy instead of placing too much emphasis only on the accuracy itself.

To speed up the CNN-based image demosaicking process, this paper proposes a compact demosaicking neural network based on UNet++. It inserts densely-connected layer blocks and adopts Gaussian smoothing layers instead of pooling layers before the backbone network to reduce the model parameters and extract mosaic image features efficiently. Furthermore, it also improves the quality of demosaicked images, and the size constraints on the input and output images are relaxed, well suitable for applications on modern edge computing devices.

## 2. Related Works

In recent years, the rapid development of hardware architectures has opened up the implementation of deep learning CNNs on modern AI image processors or other edge computing devices. More and more deep learning methods are being used in image processing. Due to the similarity between demosaicking and image super-resolution tasks, some CNN-based demosaicking methods have been gradually proposed. Inspired by SRCNN [23] and VDSR [24] for image super-resolution tasks, Syu [12] firstly proposed DMCNN and DMCNN-VD, which have similar structures with SRCNN [23] and VDSR [24], applying them to the demosaicking task. Gharbi [10] proposed a joint demosaicking and denoising (JDD) network, reaching competitive reconstruction accuracy. However, their network depends on a vast scale of datasets during training and will change the size of the output image irregularly due to their network design. In addition, there are also some algorithms for hyperspectral and multispectral image demosaicking [25,26,27], and among these, Dijkstra’s method [26] gives an effective CNN-based lightweight demosaicking method and achieves good results.

Inspired by the fact that the Bayer pattern contains twice as many green pixels as the sum of red and blue ones, Tan [11] proposed a two-stage CNN-based demosaicking network. The first stage reconstructs the intermediate estimation of the G channel, while the second stage reconstructs the R and B channels with the guidance of the reconstructed G channel. Their method achieves demosaicking images with high reconstruction accuracy but is not suitable for applications due to its complex network structure, a large number of parameters, and slow running speed. Besides, images need to be padded 48 (or other) pixels in each direction and initially recovered using bilinear interpolations before entering the network, as it is a complex pre-processing operation.

Kokkinos [13] proposed a joint demosaicking and denoising network based on a Residual Network, whose novel network structure design allows it to train a good model even on a smaller training dataset. Although the authors believe that their proposed network structure has a smaller number of parameters than other better-performing methods, a large number of cross-layer connections in their networks still makes its running time not advantageous and even longer than several other networks with a larger number of parameters. Their method requires bilinear interpolation for the input images first. So it is not suitable for application in practical situations on edge computing devices as well.

Cui [14] explored the correlation of each channel in RGB based on the method of Tan [11]’s and proposed a three-stage demosaicking network with a more complex structure, a more significant number of parameters, and the slowest running speed (compare with the other CNN-based method above), although it outperforms all previous methods. And the process also requires bilinear interpolation for the input image before processing which reduces its usefulness in practice.

## 3. Background

**Figure 1:**
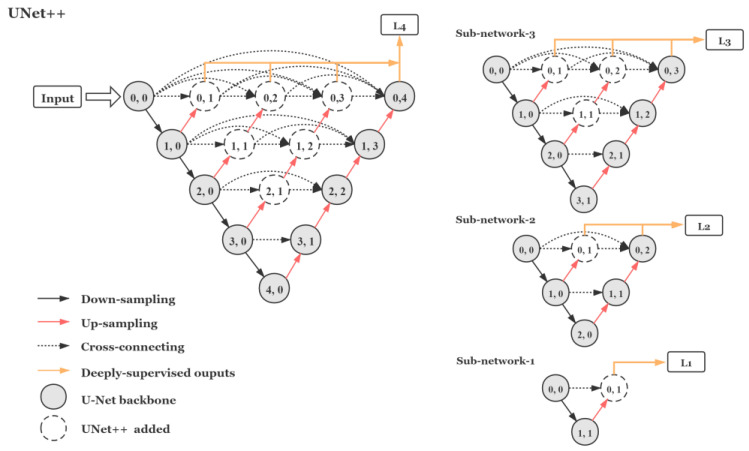
The UNet++ structure. It contains the basic U-Net backbone which is shown as gray circles in the figure. Firstly, it down-samples the input images through pooling layers at each level, then feature maps at each node get up-sampled and cross-connected with later nodes. Besides, UNet++ can be pruned during inference if trained with deep supervision. The sub-network at each level is shown on the left.

UNet++ [28] is an improved version of the U-Net [29] network structure. It is shown in an inverted pyramid shape with five levels in Figure 1. It is a deeply supervised, densely connected neural network widely used in image segmentation. It downsamples the input image layer by layer and then extracts image features through three densely connected convolutional layers. It is followed by up-sampling the corresponding number of times layer by layer until it arrives at the top and gets out. That allows image features at each level to be fused at the back end of the network. The structure of UNet++ is essentially a deeply supervised encoder–decoder network, where the encoder and decoder sub-networks are connected through a series of nested dense cross-layers.

Since UNet++ is an encoder–decoder network, it is suitable for image restoration tasks such as image denoising and defogging as well [30]. The U-Net structure has been utilized by Xie [31], Ivana [32], and some others for RGB–NIR image demosaicking tasks. As the UNet++ structure is highly flexible, we propose an end-to-end efficient demosaicking network based on it with very few parameters compared with the state-of-the-art CNN-based methods. It is capable of handling images of arbitrary size.

## 4. Proposed Network

### 4.1. Model of Image Demosaicking

The image-demosaicking problem is an optimization problem. It can be given as [33]:

(1) $$I_{raw}=MI_{rgb}+n,$$

where $I_{rgb}$ represents the ideal RGB image we aim to reconstruct, and $I_{raw}$ represents the observed sensor raw data. And M corresponds to the sampling process through the Bayer pattern, which can be represented by a square diagonal binary matrix where the zero elements in its diagonal indicate the spatial and channel locations in the image where color information is missing. And n is additive noise. Since we only focus on the process of demosaicking, we will ignore the term n. Then our optimization goal can be expressed by the following equation:

(2) $${\hat{I}}_{rgb}=DI_{raw},$$

where ${\hat{I}}_{rgb}$ denotes the estimated image reconstructed by our method, and D denotes our demosaicking process. Then the reconstruction process can be given by a minimization problem as the following equation:

(3) $$D=\arg\underset{D}{\min}\frac{1}{MN}\sum_{i}^{N}\sum_{j}^{M}\|I_{{rgb}_{i,j}}-I_{{rgb}_{i,j}}{\|}^{2},$$

the goal of this equation is to minimize the mean square error (*MSE*) of the pairs of the estimated image ${\hat{I}}_{rgb}$ and the original image $I_{rgb}$. The demosaicking task is a highly ill-posed problem because image mosaicking is non-invertible [34]. In contrast, training a neural network is essential to solving an optimization problem that satisfies the loss function through backpropagation, so it is an effective way to solve the image-demosaicking problem. Noting that the loss function chosen during training can be more than just one *MSE* loss. In this paper, the training process’s loss function is divided into two parts.

According to [35], we use *SSIM* as the loss function for the first ten epochs during training, which allows the network to learn the structural information of the image quickly:

(4) $$SSIM\left(x,y\right)=\frac{2\mu_{x}\mu_{y}+C_{1}}{\mu_{x}{}^{2}+\mu_{y}{}^{2}+C_{1}}\cdot \frac{2\sigma_{xy}+C_{2}}{\sigma_{x}{}^{2}+\sigma_{y}{}^{2}+C_{2}},$$

denotes the *SSIM* values of the corresponding pixel locations of two images. *SSIM* [36] is a metric that indicates the structural similarity of a pair of images and can represent the visual perception of human eyes to some extent. In the *SSIM* calculation process, a window of size *n* is first specified to slide over the corresponding pixels of the two images. As shown in the above equation, $\mu_{x}$, $\mu_{y}$ and $\sigma_{x}$, $\sigma_{y}$ are the mean and variance of all pixels within the sliding window; $\sigma_{xy}$ is the covariance between pixels within the window at the corresponding positions of the two images; and $C_{1}$, $C_{2}$ are constants. The larger the value of *SSIM*, the stronger the structural similarity between the two images. Then, since the optimization process of the network is the minimization of the loss function, the loss function for *SSIM* can be then written as [35]:

(5) $${ℒ}^{SSIM}=\frac{1}{MN}\sum_{j}^{N}\sum_{i}^{M}1-SSIM\left(x_{i,j}-y_{i,j}\right),$$

that is, the loss function is the average of the *SSIM* values of each pixel. The sliding window size for calculating *SSIM* is taken as 11 in our proposed network’s training process in this paper.

After ten epochs, we change the loss function to *MSE* loss so that the network can subsequently learn the image details slowly, as shown in the following equation:

(6) $${ℒ}^{MSE}=\frac{1}{MN}\sum_{j}^{N}\sum_{i}^{M}\|x_{i,j}-y_{i,j}{\|}^{2}.$$

Since our network is deeply supervised and contains three levels of outputs, the loss of *L*_1_, *L*_2,_ and *L*_3_ outputs needs to be considered together as the final loss function during training. Therefore, the final loss function of our network training is expressed as:

(7) $$ℒ=\{\begin{array}{l} {ℒ}^{SSIM}{}_{1}+{ℒ}^{SSIM}{}_{2}+{ℒ}^{SSIM}{}_{3},\;\;\;\;\;\;e\leq 10\;\\ {ℒ}^{MSE}{}_{1}+{ℒ}^{MSE}{}_{2}+{ℒ}^{MSE}{}_{3},\;\;\;\;\;\;\;\;e>10 \end{array}.$$

That is our overall optimization objective function.

### 4.2. Network Architecture

**Figure 2:**
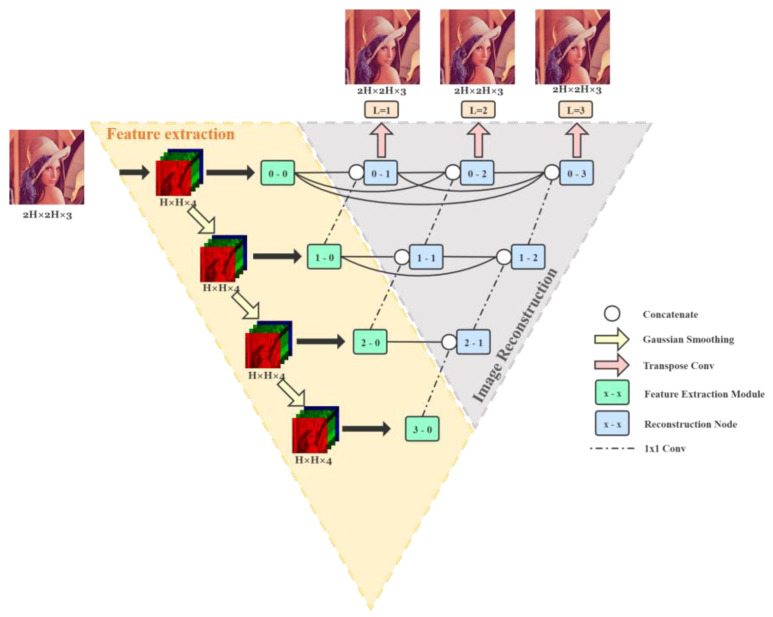
Network structure: yellow box indicates the image feature extraction part, and the gray box indicates the image reconstruction part.

Our proposed network consists of two parts: image feature extraction and image reconstruction, which are shown in Figure 2. The part boxed in yellow represents the image feature extraction part. It consists of Gaussian smoothing (the yellow arrows) at each level and feature extraction modules (the green nodes). And the part boxed in gray represents the image reconstruction part. It contains the channel concatenate operation (the white circles), the reconstruction node (the blue nodes), and the final up-sampling layer (the pink arrows).

The overall network structure is based on a deeply supervised UNet++ structure. The advantage is that when training in this deeply supervised manner, we can get outputs from different levels and can choose a sub-network from the whole model as a trade-off between the demosaicking computational costs and accuracy. Besides, the network uses Gaussian smoothing layers instead of pooling layers to expand the receptive field of the input mosaic image and keep the image size unchanged. Furthermore, it also inserts a densely-connected layer unit adopting depthwise separable convolutions at the beginning of each level to fully extract features of the mosaic image with a small number of model parameters.

Before entering the network, the input mosaic image is arranged into a four-channel pattern of RGGB and blurred separately through a Gaussian kernel for the number of times according to the level of the network at the corresponding layer. For example, in our proposed structure, the deepest level is 3, so the image will be blurred one, two, and three times and then enter the feature extraction part separately. After that, feature maps at each level will be concatenated with those from the former and lower layers and then go through the reconstruction node level by level until the top. At last, they will get out as three outputs: *L*_1_, *L*_2_, and *L*_3_.

#### 4.2.1. Image Feature Extraction

Both U-Net and UNet++ use pooling to down-sample images to obtain image features at different scales. However, for different layer levels in U-Net++, it cannot be guaranteed that an image of arbitrary size can be reconstructed to the same size as the original after several down-sampling and up-sampling operations in the image-demosaicking tasks. Therefore, in our method, we use Gaussian smoothing to process the input image with a kernel size of 5 × 5. Then we obtain a series of Gaussian pyramid-like images with the same image size and the same number of channels as the input mosaic image after each level, but with different degrees of blur. Therefore, the original down-sampling operation in the UNet++ backbone is replaced by a Gaussian smoothing layer. The advantage is that it does not change the image size, so the output and input image sizes of the network are unified while making it possible for the network to process input images of arbitrary size. More importantly, it also expands the receptive field of each input layer to fully extract features without discarding image information, unlike pooling [37]. The yellow arrows in Figure 2 show the Gaussian smoothing operation.

**Figure 3:**
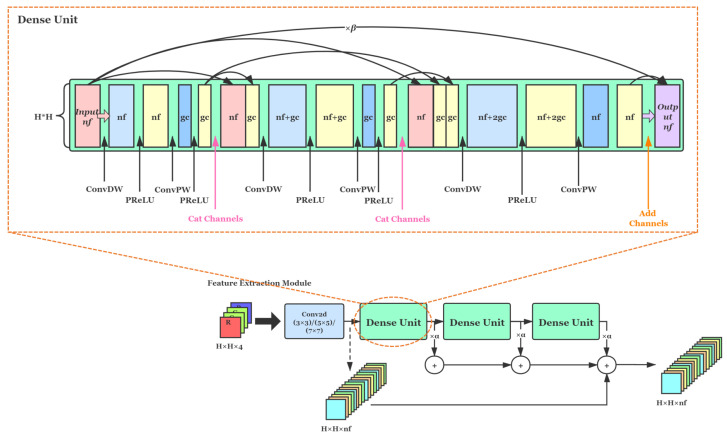
The first two cross-layer connections in each Dense Unit are connected between channels, while the cross-layer connection before the final output is summed by the feature map values. Since a normal dense block is composed of a large number of ordinary convolutions, the number of parameters could be large. Therefore, all the convolutions in our densely connected layers use depthwise separable convolutions [40], which further reduces the number of parameters of the network.

To ensure adequate extraction of image features with a sufficiently small number of network parameters, we exploited the characteristics of networks with a densely connected [38] structure, where the output feature maps at each layer are concatenated with the inputs that follow, reusing the previous feature maps. Networks can benefit a lot from this kind of structure. It can utilize the information of the image itself for self-learning only with a few network layers, and it can fully extract the high-level semantic features of the images while greatly reducing the number of parameters. As shown in Figure 3, its structure is given by a Dense Unit.

Figure 3 shows our whole structure based on the Residual in Residual Dense Block (RRDB) [39] of the feature extraction module, which contains three Dense Units, and it is still densely connected between each unit. After blurring by the Gaussian Smoothing layer, images enter the feature extraction module at each level. According to different network levels, we use a single convolution layer before three Dense Units for preliminary feature extraction, using different kernel sizes, respectively. In our method, there are 3 levels or 4 times of feature extractions. The size of kernels for layers 0, 1, 2, and 3 are set to 3 × 3, 3 × 3, 5 × 5, and 7 × 7. The purpose of applying different kernel sizes is to further expand the receptive field, enabling the network to obtain a larger range of image information near the upper layers of the image pyramid after Gaussian blur. Then feature maps will go through three Dense Units, each of which consists of three depthwise separable convolutions [40] and Parametric ReLU [41] layers after that. It is worth noting that we do not use the Batch Normalization layer in the whole structure of our method to preserve the pixel distribution characteristics of the image as well as to prevent image artifacts so that we can obtain a better recovery performance.

Noting that the Dense Unit is characterized by the same number of input channels as the number of output channels. Other optional parameters are the number of input channels *nf* and the number of intermediate channels *gc*. The values of the parameters *nf* and *gc* used in our training process at each level are given in Table 1.

#### 4.2.2. Image Reconstruction

After the feature extraction part, the features of each level are then concatenated with the results of the former and lower layers. It will go rightward and upward until the top and get out hierarchically. That forms the image construction part of the network.

The reconstruction part is still densely cross-connected, like UNet++. The blue node in Figure 2 represents each reconstructing operation unit, consisting of a 3 × 3 convolutional layer, a 1 × 1 convolutional layer, and a Parametric ReLU layer. The white circle shown in Figure 2 represents the concatenation of input feature maps over channels; and the dashed arrow indicates the up-sampling operation between layers, using a convolution with a kernel size of 1 × 1; and the pink arrow indicates the up-sampling process at the end of the network before the final output of the reconstructed image, using a transposed convolution with a kernel size of 3 × 3. After that, it gives reconstructed images to three different levels.

The input image size and the size of the feature map inside the network at each level keep the same until final up-sampling, after which it is two times the original height and width. The details of feature map size and kernel size at each reconstruction node during the network are shown in Table 2.

During the training process, we add all three levels of outputs to the loss function to provide feedback to the network, and the sub-network of different layers can be taken out separately during the inference process, making it flexible in choosing a different scale of the network to suit different application needs. The higher the level of the network output, the higher the demosaicking accuracy, and the larger the number of parameters.

## 5. Experiments and Results

### 5.1. Training

With a small number of network parameters, the selection of a suitable training set becomes more important for the training process. To have a better reconstruction performance on various kinds of scenes, we choose ImageNet [42], which contains 130,000 images composed of 1000 classes of objects as the training dataset. It contains a large number of images with different scenes and structures, which helps the network to learn the reconstruction process for images with different color distributions and scenes. We first crop a 256 × 256 image from the center of each original image in the training set and then divide each one into four 128 × 128 patches, for a total of 520,000 patches as our training set.

**Figure 4:**
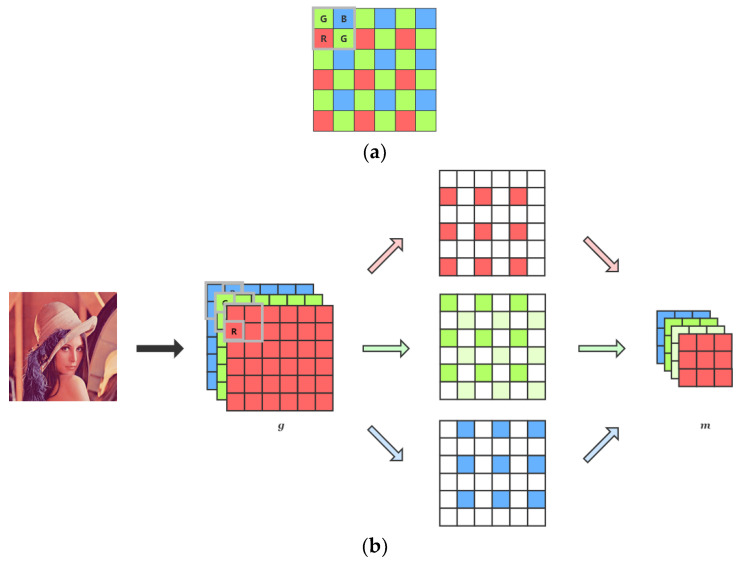
(**a**) The channel (or color) position to be chosen in a 2 × 2 window. (**b**) The process of generating network input from an original RGB image. Note that the two green color of different intensities in the middle of (**b**) both represents the green channel. Just to make the image-generating process more clear and understandable, we painted it to the different green intensities.

We use one of the Bayer pattern GBRG as the filter to produce mosaic patches. As shown in Figure 4, we first take one channel from each position of the RGB images in a 2 × 2 window according to the arrangement of the GBRG pattern, and then we arrange them in the order of R, G, G, and B to form a four-channel image with the shape of 64 × 64 × 4 as the network input *m*. The output *o* of the network is of the same size as the original ground-truth RGB patch, which is 128 × 128 × 3.

As for training settings, we adopt Adam [43] as the optimizer; the initial learning rate is set to 0.001. It drops to half by a factor of every 10 epochs. The number of training minibatches is set to 16. For the other hyper-parameters of Adam, we use the default setting. As given in Section 4.1, we use SSIM as the loss function during the first 10 epochs and later use MSE as the loss function. The training process is carried out on Nvidia GeForce RTX 3090 on Windows 10 64-bit OS with Intel i5-9400F (2.9 GHz) processor with 150 epochs, and we finally took the best performance one for later testing.

### 5.2. Tests for Quantitative and Qualitative Performance

For the demosaicking task, there are two most widely used datasets, Kodak [44] and McMaster [45], in addition to which we add Urban100 [46], a dataset containing 100 buildings images with stripes and lattice features, and Mange109 [47], a dataset containing 109 colored comics, for a total of 251 images for testing. These four datasets cover most scenarios as well as the challenging features of image-demosaicking tasks. Note that we will use Kod, McM, Urb, and Man to denote the abbreviated form of those datasets respectively in the below result tables. We selected the following traditional demosaicking methods: AHD [48], DLMMSE [49], RI [5], MLRI [6], ARI [7], as well as the three CNN-based demosaicking methods: Tan [11], Kokkinos [13], and Cui [14] mentioned in Section 2 for comparison, all using the open-source code on their project home page. The five traditional methods are given by Matlab code, and CNN-based ones are given by Python. We use the peak signal-to-noise ratio (*PSNR*) value, or the color peak signal-to-noise ratio (*CPSNR*) value as the metric to measure the reconstruction accuracy of the demosaicked image, as defined below [50]:

(8) $$CPSNR=10\;\log_{10}\frac{255^{2}}{\frac{1}{3}\sum_{R,G,B}\frac{1}{MN}\sum_{j}^{N}\sum_{i}^{M}\|x_{i,j}-y_{i,j}{\|}^{2}}.$$

Each algorithm was tested on four datasets to get the *PSNR*s (for each single color) and the *CPSNR*s (for the whole image of three colors). Due to their difference in padding and other pre-processing strategies, a 5-pixel at each edge was ignored when testing the *PSNR* values. The results are shown in Table 3.

**Figure 5:**
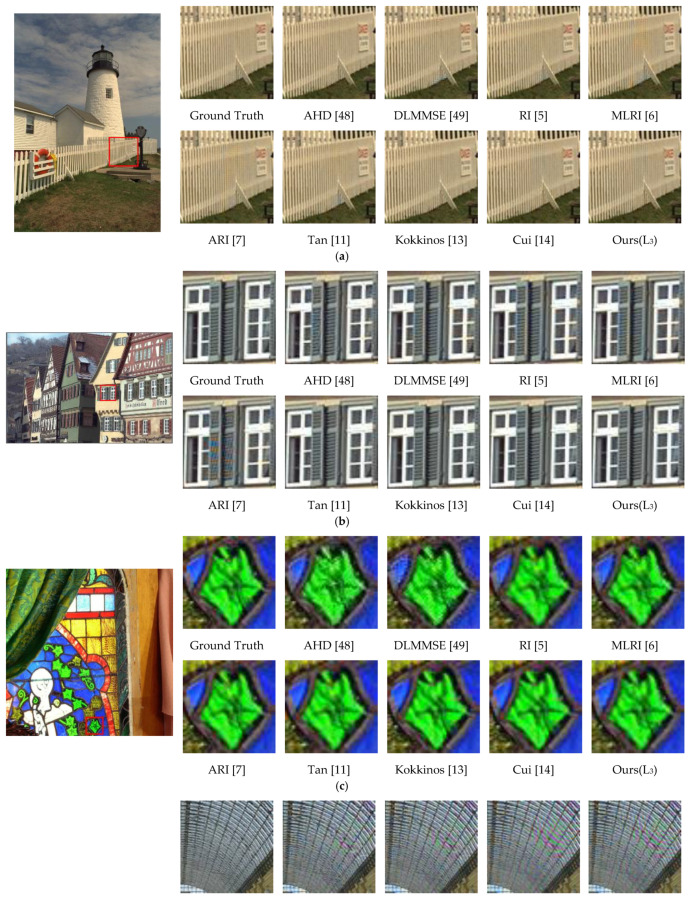

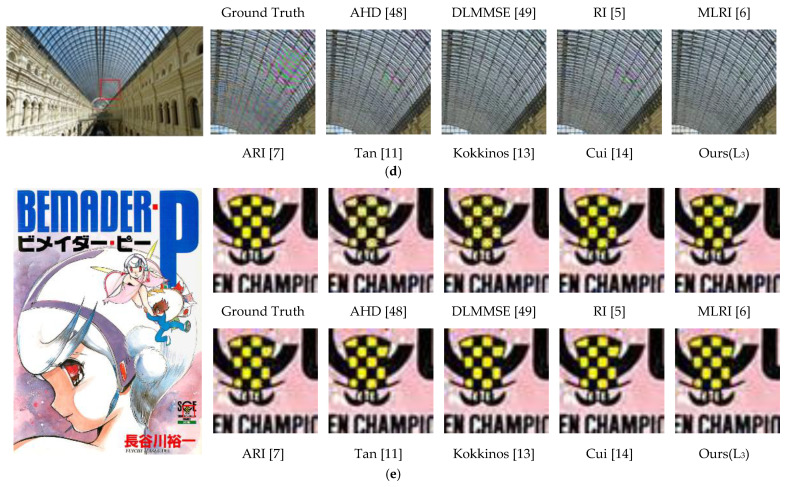
Visual comparison of the demosaicking results on 5 representative images in Kodak, McMaster, Urban100, and Manga109. (**a**) ‘kodim19’ from Kodak [44]. (**b**) ‘kodim08‘ from Kodak [44]. (**c**) ‘1’ from McMaster [45]. (**d**) ‘img_008’ from Urban100 [46]. (**e**) ‘BEMADER_P’ from Manga109 [47].

From the table, we can see that the performance of the CNN-based methods is substantially better than that of the traditional ones in terms of the *PSNR* metric. Although the proposed method does not reach the highest *PSNR* value, it is among top-level, indicating that it can reconstruct sufficient high-quality images. Meanwhile, we selected several images with some representative as well as difficult structures from those datasets to give the visual reconstruction comparison in Figure 5.

From the visual comparison of demosaicking results above, we can see that AHD [48] has a better demosaicking visual performance in those traditional methods, while the performance of CNN-based methods is better than the traditional ones overall. At the same time, the proposed method in this paper results in a very good visual performance in all kinds of patches. Even when Cui [14] and Kokkinos [13] achieve top *PSNR*s in Table 3, introducing some pink artifacts in Figure 5d, the results of our method produce no artifacts. As a result, although the reconstruction *PSNR* value of our method is not the highest, it still shows an excellent performance in highly difficult demosaicking scenes such as stripes without generating artifacts like zippering and false color.

### 5.3. Tests for Computational Cost

Image-demosaicking is an important part of the modern camera ISP pipeline. The reconstruction accuracy of the demosaicking method is important, but blindly pursuing high *PSNR* while ignoring the computational cost loses its meaning for practical application. As mentioned in [22], current works on demosaicking have achieved top demosaicking accuracy on benchmark datasets which is high enough. So running speed and memory required to store model parameters (especially for the CNN-based method) are the main issues that should be considered first.

We measured the running time required on a total of 18 images of the shape 500 × 500 in McMaster datasets [11] on the Nvidia AGX Xavier (32 GB) AI accelerator and calculated the average processing time of each image. Nvidia AGX Xavier is an embedded edge device that can efficiently perform parallel computations through an edge GPU. It is a promising platform for embedded machine learning [51]. Tests were carried out while all other processes in the device were turned off. Besides, for each CNN-based method, the number of parameters was counted to obtain the storage space required for double-precision floating-point format parameters of the models. The results are shown in Table 4.

Due to the differences in the generation and pre-processing between each method for the input mosaic images, the time we measured above includes the process from the original RGB image to the generation of mosaic images. Each method was tested five times and averaged as the final result. Among them, the traditional method, AHD [48], DLMMSE [49], RI [5], MLRI [6], and ARI [7], are originally implemented in Matlab. To deploy them to the Nvidia AGX Xavier platform, we rewrote those algorithms in Python without changing their structure and computational details. Then, in order to accelerate them through CUDA, we modified them using the cupy library, which professionally accelerates computations for numpy matrices on the GPU.

As for Tan [11], Kokkinos [13], Cui [14], and our proposed method, all of them are implemented by the CNN approach, and thus we accelerated them using pytorch on CUDA.

**Figure 6:**
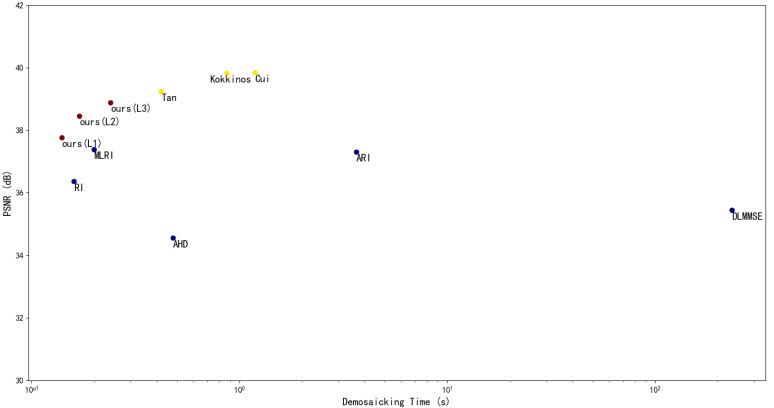
The scatter plot of *PSNR*s and the running time of log scale on the 9 demosaicking methods.

As shown in Table 4, the method proposed in this paper has apparent advantages in terms of running time and the number of parameters. Their trade-off between *PSNR*s and running time is illustrated by the scatter in Figure 6 (the time axis is of log scale rather than linear scale due to the big gap between running times of some methods).

We can see from the plot that none of the methods above outperforms our proposed method by both higher *PSNR* and lower time cost, whereas the proposed method (L_3_) outperforms five out of the eight methods by both *PSNR* and running time. Compared with Kokkinos [13] and Cui [14], which achieve the highest *PSNR*, the proposed method only loses 2.37% and 2.43% of the *PSNR* value while improving the running time by 72.18% and 79.59%. Compared with the fastest method in CNN-based methods Tan [11], the proposed method can improve the running speed by 42%, with an accuracy loss of just 0.93%.

### 5.4. Tests for Extended Applications

Considering the wide application of edge computing devices for image classification and detection tasks, we performed each demosaicking method before the image classification and detection tasks, simulating the process of outputting mosaic images from image sensors, going through demosaicking algorithms, and then passing through classification or detection networks. We tested the accuracy of classification and detection tasks after different demosaicking algorithms and compared the reconstruction effect. We selected MobileNet v1 [40] and SSD [52] as the algorithms for image classification and image target detection and selected the test sets from ImageNet [42] (1000 classes with 50 images per class, totally 50,000 images) and PASCAL VOC2007 (totally 4952 images of 20 categories), respectively, as the test sets. We used the official pre-trained models for MobileNet v1 on ImageNet and SSD on VOC2007.

To the image classification task on MobileNet v1, for each original RGB image in the test set, the corresponding reconstructed image was generated first according to the demosaicking method mentioned in Section 5.2. Then the images were uniformly cropped from the center of the original images to 224 × 224 and got classified by the pre-trained MobileNet v1 model. Finally, the Top-1 and Top-5 classification accuracy on 1000 classes was calculated.

To the target detection task, we first took the same operation for each input RGB image in the VOC2007 test set as the image classification part to generate the reconstruction images. After that, each image was resized to 300 × 300 and then entered SSD for detection. Finally, the average detection accuracy (mAP value [53]) over the 20 categories was calculated.

We list the accuracy values of each demosaicking method after connected with the image classification and detection tasks in Table 5.

As shown in Table 5, after reconstruction through different demosaicking methods, there is some degree of loss in accuracy for both classification and detection tasks, but the difference between them is small. As a result, our proposed method and Tan’s [11] get the best performance among all the algorithms in image classification tasks.

### 5.5. Ablation Study for the Gaussian Smoothing Layers

In this section, we add an ablation study for the Gaussian smoothing layers and discuss its impact in the proposed network. Table 6 gives a comparison of the demosaicking accuracy obtained on the four datasets through networks with different structures, which replacing the Gaussian smoothing layers with pooling layers in different manners. The data in Table 6 were testing after the networks trained to convergence.

## 6. Conclusions

In this study, we propose a compact, high-efficiency end-to-end demosaicking convolutional neural network for the current application needs on edge computing devices. By adding densely connected layer blocks and using depthwise separable convolutions, we made full use of the correlations between the features of the images themselves for computation, which greatly reduced the number of parameters of the network but still achieved excellent performance. Besides, we used Gaussian smoothing instead of down-sampling input images to expand the receptive field and to relax the constraints on the size of input images without discard any image information. Furthermore, since Gaussian smoothing can play a certain degree of denoising, and its use leads the network to be able to extract multi-scale image features, the proposed network has the potential to perform well in other image restoration tasks such as denoising and super-resolution. We will apply the proposed network structure to these tasks in future research.

The above experiment results demonstrated that our proposed method achieves the leading demosaicking accuracy in terms of both subjective visual comparisons and the objective metric (*PSNR*). Besides, the result of inference processing on the demosaicked images on Mobilenet v1 and SSD indicates that the accuracy can also achieve a high level that is performed comparably to the existing methods. Moreover, it contains only several convolutional computations with high parallelism and can handle images of arbitrary size without special pre-processing operations for the input mosaic images, making it easily connected with image sensors with a CFA pattern. Its deeply supervised training manner makes it flexible to be pruned during inference. As a result, the proposed methods can be efficiently applied to some edge computing devices such as AI accelerators and has the potential for efficient processing on edge devices that support parallel processing such as Application Specific Integrated Circuit (ASIC) or intelligent camera processors for high-quality image demosaicking.

## Figures and Tables

**Table 1:** Details of the parameters *nf* and *gc* in each Dense Unit used in our network.

Node	nf	gc
0–0	8	4
1–0	16	8
2–0	32	16
3–0	64	32

**Table 2:** Network details of each node in Figure 2, including the convolutional kernel size of the reconstruction nodes, the input size, and the output size of feature maps.

Node	Kernel Size	Input Size	Output Size
0–0	-	H × W × 4	H × W × 8
0–1	3 × 3 × 16 × 16; 1 × 1 × 16 × 8	H × W × 16	H × W × 8
0–2	3 × 3 × 16 × 16; 1 × 1 × 16 × 8	H × W × 16	H × W × 8
0–3	3 × 3 × 16 × 16; 1 × 1 × 16 × 8	H × W × 16	H × W × 8
1–0	-	H × W × 4	H × W × 16
1–1	3 × 3 × 32 × 32; 1 × 1 × 32 × 16	H × W × 32	H × W × 16
1–2	3 × 3 × 32 × 32; 1 × 1 × 32 × 16	H × W × 32	H × W × 16
2–0	-	H × W × 4	H × W × 32
2–1	3 × 3 × 64 × 64; 1 × 1 × 64 × 32	H × W × 64	H × W × 32
3–0	-	H × W × 4	H × W × 64

**Table 3:** The *PSNR* values (dB) of each channel (R, G, B) and *CPSNR*s of the whole image (RGB) of 9 demosaicking algorithms on Kodak, McMaster, Urban100, Manga109 datasets and the final average values of them. The bolded two methods in each section are those that achieve better performance among all algorithms.

Algorithm		AHD [48]	DLMMSE [49]	RI [5]	MLRI [6]	ARI [7]	Tan [11]	Kok ^1^ [13]	Cui [14]	Ours(L_1_)	Ours(L_2_)	Ours(L_3_)
Kod	R	36.88	38.47	37.83	38.87	39.11	41.11	41.30	41.98	40.29	40.88	41.22
	G	39.59	42.65	41.03	41.86	42.33	44.86	45.96	45.10	43.44	44.00	44.34
	B	37.37	38.53	37.80	38.86	38.77	40.80	41.29	41.04	39.68	40.24	40.59
	RGB	37.74	39.36	38.57	39.58	39.75	41.82	**41.96**	**42.32**	40.82	41.38	41.72
McM	R	33.01	33.13	36.12	36.38	37.44	38.54	39.93	39.70	36.64	37.56	38.01
	G	36.99	38.00	40.00	39.91	40.73	41.95	42.65	42.63	39.61	40.31	40.74
	B	32.16	31.84	35.37	35.38	36.07	37.14	38.00	37.72	35.25	35.90	36.33
	RGB	33.50	33.55	36.50	36.65	37.54	38.67	**39.67**	**39.45**	36.75	37.48	37.91
Urb	R	32.63	33.91	33.72	36.38	34.63	37.14	38.68	37.71	35.54	36.30	36.84
	G	35.62	37.65	36.67	39.91	38.03	40.94	42.33	41.42	39.31	39.99	40.46
	B	32.87	33.92	33.90	35.38	34.79	37.13	38.54	37.70	35.59	36.30	36.90
	RGB	33.42	34.73	34.48	36.65	35.49	38.00	**39.47**	**38.56**	36.44	37.16	37.70
Man	R	32.01	32.71	34.68	36.38	35.58	37.31	38.00	38.16	36.11	36.90	37.27
	G	38.14	39.45	40.31	39.91	40.30	43.23	43.35	43.60	40.55	41.36	41.93
	B	33.10	33.23	35.10	35.38	35.34	37.37	36.16	37.68	35.97	36.62	37.01
	RGB	33.55	34.11	35.88	36.65	36.43	**38.47**	38.17	**39.05**	37.04	37.77	38.17
Ave.	R	33.63	34.55	35.59	37.00	36.69	38.53	39.48	39.39	37.14	37.91	38.33
	G	37.59	39.44	39.50	40.40	40.35	42.75	43.57	43.19	40.73	41.41	41.87
	B	33.88	34.38	35.54	36.25	36.24	38.11	38.50	38.54	36.62	37.27	37.71
	RGB	34.55	35.44	36.36	37.38	37.30	39.24	**39.82**	**39.84**	37.76	38.45	38.88

^1^ Due to the limitations of the table format, we abbreviate ‘Kokkinos’ to ‘Kok’.

**Table 4:** The average running time of a 500 × 500 image in McMaster and the number and size (for double-precision floating-point format) of parameters for CNN-based models. The bolded two methods in each section are those that achieve better performance among all algorithms.

Algorithm	Running Time (s)	Parameters	
		Number	Size (MB)
AHD [48]	0.48	-	-
DLMMSE [49]	234.78	-	-
RI [5]	**0.16**	-	-
MLRI [6]	0.20	-	-
ARI [7]	3.66	-	-
Tan [11]	0.42	528,518	2.02
Kokkinos [13]	0.87	380,356	1.45
Cui [14]	1.19	1,793,032	6.84
Ours (L_1_)	**0.14**	**11,786**	0.04
Ours (L_2_)	0.17	**46,537**	0.18
Ours (L_3_)	0.24	183,628	0.70

**Table 5:** The accuracy of classification and detection tasks connected with demosaicking methods. The ‘Origin’ item indicates the original accuracy of the pre-trained model MobileNet v1 and SSD300. The bolded two methods in each section are those that achieve better performance among all algorithms.

Algorithm	MobileNet v1		SSD300
	Top1 (%)	Top5 (%)	mAP (%)
Origin	71.11	89.84	75.77
AHD [48]	**64.79**	85.67	75.41
DLMMSE [49]	64.06	85.44	75.14
RI [5]	64.25	85.65	75.16
MLRI [6]	64.36	85.70	75.21
ARI [7]	64.40	85.74	75.06
Tan [11]	**65.02**	**86.04**	**75.59**
Kokkinos [13]	64.43	85.76	**75.56**
Cui [14]	64.50	85.80	75.49
Ours (L_1_)	64.11	85.49	75.16
Ours (L_2_)	64.43	85.78	75.22
Ours (L_3_)	64.56	**85.83**	75.44

**Table 6:** Testing results on four datasets for network structures with different pooling layers. Only the results for the L_3_ network are presented here for clearer comparisons of different network structures.

Algorithms		Avg Pooling	Max Pooling	Gaussian Pooling	Gaussian Smoothing
		L = 3	L = 3	L = 3	L = 3
Kodak24	R	40.95	40.74	40.86	41.22
	G	44.09	43.86	44.01	44.34
	B	40.35	40.11	40.28	40.59
	RGB	41.47	41.25	41.39	**41.72**
McMaster	R	37.83	37.79	37.71	38.01
	G	40.58	40.53	40.56	40.74
	B	36.07	36.03	35.97	36.33
	RGB	37.70	37.66	37.61	**37.91**
Urban100	R	36.67	36.34	36.58	36.84
	G	40.29	39.99	40.22	40.46
	B	36.68	36.32	36.56	36.90
	RGB	37.51	37.17	37.41	**37.70**
Manga109	R	36.93	36.72	36.90	37.27
	G	41.53	41.30	41.54	41.93
	B	36.75	36.54	36.74	37.01
	RGB	37.86	37.65	37.84	**38.17**
Ave.	R	38.10	37.90	38.01	38.33
	G	41.62	41.42	41.58	41.87
	B	37.46	37.25	37.39	37.71
	RGB	38.64	38.43	38.56	**38.88**

Where Avg pooling, Max pooling, Gaussian pooling, and Gaussian smoothing denote 2 × 2 average pooling, 2 × 2 max pooling, Gaussian smoothing followed by 2 × 2 down-sampling, and Gaussian smoothing layer (used in this paper), respectively. Since pooling changes the image size, in each image reconstruction node in the original network, we replace the 1 × 1 convolution with up-sampling implemented by a transposed convolution. It can be seen that the network adopting Gaussian smoothing layers can extract image features more efficiently than other pooling approaches, thus obtaining a little better accuracy due to its capability to extract image features through multi-scale receptive fields and its preservation of entire image information.

## Data Availability

Publicly available datasets were analyzed in this study. The data can be found here: (ImageNet2012) https://image-net.org/download.php; (Kodak24) http://r0k.us/graphics/kodak/; (McMaster) https://www4.comp.polyu.edu.hk/~cslzhang/CDM_Dataset.htm; (Urban100) https://github.com/jbhuang0604/SelfExSR; (Manga109) http://www.manga109.org/en/download.html.
